# Whole genome bisulfite sequencing reveals DNA methylation roles in the adaptive response of wildness training giant pandas to wild environment

**DOI:** 10.3389/fgene.2022.995700

**Published:** 2022-10-11

**Authors:** Xiaodie Jie, Honglin Wu, Miao Yang, Ming He, Guangqing Zhao, Shanshan Ling, Yan Huang, Bisong Yue, Nan Yang, Xiuyue Zhang

**Affiliations:** ^1^ Key Laboratory of Bio-resources and Eco-environment, Ministry of Education, College of Life Science, Sichuan University, Chengdu, China; ^2^ Key Laboratory of State Forestry and Grassland Administration on Conservation Biology of Rare Animals in the Giant Panda National Park, China Conservation and Research Center for the Giant Panda, Dujiangyan, China; ^3^ Institute of Qinghai-Tibetan Plateau, Southwest Minzu University, Chengdu, China; ^4^ Sichuan Key Laboratory of Conservation Biology on Endangered Wildlife, College of Life Sciences, Sichuan University, Chengdu, China

**Keywords:** giant panda, wildness training, whole genome bisulfite sequencing, adaptive response, immunity

## Abstract

DNA methylation modification can regulate gene expression without changing the genome sequence, which helps organisms to rapidly adapt to new environments. However, few studies have been reported in non-model mammals. Giant panda (*Ailuropoda melanoleuca*) is a flagship species for global biodiversity conservation. Wildness and reintroduction of giant pandas are the important content of giant pandas’ protection. However, it is unclear how wildness training affects the epigenetics of giant pandas, and we lack the means to assess the adaptive capacity of wildness training giant pandas. We comparatively analyzed genome-level methylation differences in captive giant pandas with and without wildness training to determine whether methylation modification played a role in the adaptive response of wildness training pandas. The whole genome DNA methylation sequencing results showed that genomic cytosine methylation ratio of all samples was 5.35%–5.49%, and the methylation ratio of the CpG site was the highest. Differential methylation analysis identified 544 differentially methylated genes (DMGs). The results of KEGG pathway enrichment of DMGs showed that *VAV3*, *PLCG2*, *TEC* and *PTPRC* participated in multiple immune-related pathways, and may participate in the immune response of wildness training giant pandas by regulating adaptive immune cells. A large number of DMGs enriched in GO terms may also be related to the regulation of immune activation during wildness training of giant pandas. Promoter differentially methylation analysis identified 1,199 genes with differential methylation at promoter regions. Genes with low methylation level at promoter regions and high expression such as, *CCL5*, *P2Y13*, *GZMA*, *ANP32A*, *VWF*, *MYOZ1*, *NME7*, *MRPS31* and *TPM1* were important in environmental adaptation for wildness training giant pandas. The methylation and expression patterns of these genes indicated that wildness training giant pandas have strong immunity, blood coagulation, athletic abilities and disease resistance. The adaptive response of giant pandas undergoing wildness training may be regulated by their negatively related promoter methylation. We are the first to describe the DNA methylation profile of giant panda blood tissue and our results indicated methylation modification is involved in the adaptation of captive giant pandas when undergoing wildness training. Our study also provided potential monitoring indicators for the successful reintroduction of valuable and threatened animals to the wild.

## 1 Introduction

External factor changes leads to epigenetic modification of organisms, and this will mediate the interaction between the environment and genes, which is conducive to the adaptation of organisms to a new environment (Tammen et al., 2013). DNA methylation can regulate gene expression without changing the genome (Goldberg et al., 2007). It is the most important epigenetic modification of organisms, and it plays an important role in the rapid environmental adaptation of organisms (Waterland, 2006; Anderson et al., 2012; Liu, 2013; Huang et al., 2017; Thiebaut et al., 2019). For example, methylation modification can affect the phenotypic variation of worker bees and the cold stress and heat response of fish, thereby increasing population adaptability (Shi et al., 2011; Han et al., 2016; Anastasiadi et al., 2017). Marbled Crayfish, Anolis Lizards, and other species can rapidly respond to acute environmental changes through DNA methylation modifications (Huang et al., 2017; Hu et al., 2019; Tönges et al., 2021). Prenatal exposures to air pollutants in the first trimester could influence placental adaptation by DNA methylation (Maghbooli et al., 2018). DNA methylation has also an important role in the response of three-spined stickleback fish to parasitic infection (Sagonas et al., 2020). Despite the certain understanding of the methylation patterns in the above animals, there is a paucity of studies on methylation modifications in non-model mammals, such as giant pandas.

The giant panda is the “national treasure” of China, a first-grade state protected animal and IUCN Vulnerable Animal with a global reputation. The giant panda is seriously threatened due to habitat fragmentation, human population growth and climate change. Recent conservation efforts in China have focused on minimizing threats and thus there have been moderate improvements in the wild giant panda population, which has been alongside a successful captive breeding program to ensure a reserve genetically diverse population (Wei et al., 2015; Xiaoping et al., 2015; Martin-Wintle et al., 2019). However, several of the 33 local wild giant panda populations are significantly threatened with extinction and conservation efforts have been unsuccessful (State Forestry Administration, 2015). Wildness and reintroduction of giant pandas are the important content of giant panda conservation at present and in the future (Zhang et al., 2006). The captive and wild environments significantly differ. The living conditions of captive giant pandas are small, confined, their diet is closely monitored and the pens are disinfected, but they are safe from threats, have few stressors and live longer than wild conspecifics. Wild giant pandas can move relatively freely in their diverse habitat and their diet can be varied, yet they are not protected from threats and thus they generally have increased stressors and injuries and shortened lifespans. Consequently, it is often difficult for released individuals to adapt to the wild environment, causing reintroduction efforts to fail (Allard et al., 2019). The released individuals must gradually establish an adaptive mechanism to cope with changes from captivity to the wild. Therefore, it is necessary to conduct wildness training for giant pandas to improve their ability to adapt to the wild before they are finally released. However, it is unclear how wildness training affects the epigenetics of giant pandas, and we lack the means to assess the effectiveness of wildlife training in giant pandas.

DNA methylation is the most important epigenetic modification in organisms, and changes in it often lead to alterations in gene expression. In addition, combined analysis of DNA methylation and gene expression has been extensively used to provide an in-depth understanding of growth and development, disease occurrence, and environmental adaptation processes (Shi et al., 2020). Therefore, the study of DNA methylation before and after wildlife training of giant pandas can be helpful in providing some insights into the establishment of indicators for assessing the effectiveness of wildlife training of giant pandas. Bisulfite sequencing can obtain biological single-base-resolution DNA methylation profiles, and is currently the most accurate and efficient method for depicting genome-wide DNA methylation profiles. In this study, Whole Genome Bisulfite Sequencing (WGBS) was used to analyze genomic methylation differences between captive giant pandas with (“wildness training” group) and without (“captive” group) wildness training to understand the characteristics of DNA methylation modifications from their wild training. We also aimed to clarify the regulatory role of DNA methylation modifications in the environmental adaptation of giant pandas in combination with transcriptome data.

## 2 Materials and methods

### 2.1 Collection and processing of blood samples from giant pandas

Giant panda blood samples were provided by Chengdu Research Base of Giant Panda Breeding in Chengdu and China Research and Conservation Center for the Giant Panda at Dujiangyan, Sichuan Province, China, and were collected by veterinarians during routine examinations of giant pandas (Supplementary Table S1). The sample collection was approved by the ethics committee of the College of Life Sciences, Sichuan University (Grant No: 20190506001), and the experimental procedures were in accordance with Chinese regulations on animal welfare and related laws. Six WGBS samples and twelve transcriptome sequencing (RNA-seq) samples were obtained from fifteen giant pandas (seven in the wild training group and eight in the captive group). Twelve RNA-seq samples have been described in our previous study (Yang et al., 2022).

### 2.2 Library preparation and sequencing

Library preparation, sequencing and data analysis of RNA-seq samples have been described in our previous study (Yang et al., 2022). All RNA-seq data have been submitted to NCBI Sequence Read Archive with BioProject numbers PRJNA878951.

The library preparation and sequencing process for WGBS samples is as follows. The DNeasy Blood & Tissue Kit (TIANGEN, Beijing, China) was used to extract genomic DNA from giant panda blood tissues according to the instructions. After checking the DNA purity and concentration, WGBS was performed. The sequencing was performed by Novogene Bioinformatics Institute (Beijing, China) for PE150 sequencing. The sequencing platform was Illumina NovaSeq 6000 (Illumina, Inc., San Diego, CA, United States). We used EZ DNA Methylation-GOLD^TM^ Kit (ZYMO-RESEARCH, CA, United States) for bisulfite conversion of whole genome DNA. In this process, bisulfite converted the cytosine in the DNA sequence into uracil, while methylated cytosine was not affected. After base conversion, PCR amplification was performed to obtain the final DNA methylation sequencing library. In this step, due to PCR amplification, uracil continued to be converted to thymine, thereby completing the conversion process. After the library was constructed, Qubit 2.0, Agilent 2100 and quantitative PCR were used to perform accurate quantification and integrity detection of the constructed library. After the library was qualified, the Illumina NovaSeq sequencing was performed to obtain the sequence information for the subsequent analysis. All WGBS data have been submitted to NCBI Sequence Read Archive with BioProject numbers PRJNA857106.

### 2.3 WGBS sequencing data analysis

Clean reads of WGBS sequencing data were applied to the reference genome of the giant panda with Bismark software (Krueger and Andrews, 2011) (version 0.22.3). The reference genome and annotation files were downloaded from the Ensembl database (http://asia.ensembl.org/index.html). In the process of methylation sequencing, the C base (unmethylated) is converted to T base due to the treatment of bisulfite. Therefore, in the process of using Bismark software for comparison, it was also necessary to convert all C bases of reads to T bases (the positive chain was converted from C bases to T bases, and the reverse chain was converted from G bases to A bases), and then we judged whether the position had undergone methylation modification according to the comparison result. We used the bismark_genome_preparation function to construct the index after transforming the same reference genome, and then we called bowtie2 to compare the reads to the reference genome. The comparison parameters were set to: N 1 -L 20 --score_min L, 0,-0.2 --bowtie2.

### 2.4 Methylation site extraction

Since repetitive sequences were generated during the PCR amplification process, the data was deduplicated before the methylation sites extracted. We used the deduplicate_bismark function of Bismark software to delete the duplicate data. After deduplication, we used the bismark_methylation_extractor function of Bismark software to extract methylation sites. Bismark extraction results contained the methylation site information in three sequence environments. After subsequently counting the cytosine methylation sites, we mainly focused on the methylation sites in the CG sequence environment.

### 2.5 Differential methylation analysis

The DSS software package (Feng et al., 2014) (version 2.34.0) in R software (version 3.6.1) was used to extract differentially methylated regions (DMRs). DMRs refer to the methylation level of genomic regions with statistically significant differences between the wildness training group and the captive group. DSS calculated the average methylation level and dispersion degree of all CpG sites, and performed the Wald test to find DMRs based on the β-negative binomial distribution model. We set the parameters of DSS to: p. threshold = 0.05, delta = 0.1, smoothing = TRUE, and other parameters were set as default. We used the findOverlaps function of the GenomicRanges software package (Lawrence et al., 2013) (version 1.38.0) in the R software to annotate the obtained DMRs. DMRs were annotated to the part of genes ranging from 1 kb upstream of the TSS to 1 kb downstream of the TTS were define as DMGs.

### 2.6 GO and KEGG enrichment analysis of DMGs

GO and KEGG enrichment analyses were performed using g:Profiler (http://biit.cs.ut.ee/gprofiler/gost) and KOBAS (http://kobas.cbi.pku.edu.cn/kobas3) online software, respectively. The giant panda genome was selected as the background gene set. Pathways with more than two enriched genes and *p* less than 0.05 were identified as significantly enriched pathways.

### 2.7 Differentially methylated promoter identification

A promoter is an important sequence that can regulate gene expression, and the methylation modification of the promoter usually leads to gene transcription silencing, thereby negatively regulating gene expression (Deaton and Bird, 2011; Moore et al., 2013). We calculated the promoter methylation levels of all genes in six samples (only analyzed the methylation levels in the CG sequence environment) to determine whether there was a difference in the methylation modification of the promoter region between the wildness training and captive groups. Among them, we defined the 1 kb range upstream of the TSS as the promoter region of the gene, and it needed to contain more than two methylation sites. The Wilcoxon rank sum test of one-tailed was used, and the significance threshold was set to 0.05.

### 2.8 Correlation analysis of differentially methylated promoter and gene expression

After identifying a set of genes undergoing methylation changes in promoter region between the wildness training and captive groups, we combined RNA-seq data from wildness training and captive pandas to assess the associated changes in gene expression and promoter methylation levels (Yang et al., 2022). We investigated the correlation between changes in methylation levels of promoter region and gene expression levels by Spearman rank correlation analysis with a two-tailed t-test.

## 3 Results

### 3.1 Whole genome methylation sequencing

Whole genome methylation sequencing obtained 349.72G WGBS data in the wildness training group and 369.86G in the captive group. The bisulfite conversion rate (the ratio of bisulfite converting C bases to T bases) of all samples was greater than 99%. After removing the adapters and low-quality sequences, the remaining clean reads was: wildness training group 341.74G, captive group 358.02G (Table 1).

**TABLE 1:** The methylation sequencing quality and genome coverage of WGBS samples in this study.

Sample ID	Sex	Group	Raw base(G)	Clean base(G)	BS Conversion Rate (%)	Mapping Rate (%)
QX	Female	Wildness training	120.8	119.72	99.21	72.20
XHT	Female	Wildness training	117.26	116.00	99.14	71.70
RR	Male	Wildness training	111.66	106.02	99.11	66.95
PQ	Female	Captive	123.34	121.42	99.27	73.70
LL	Female	Captive	123.16	120.78	99.28	72.80
QL	Male	Captive	123.36	115.82	99.14	59.67

### 3.2 Distribution and statistics of cytosine methylation

**FIGURE 1:**
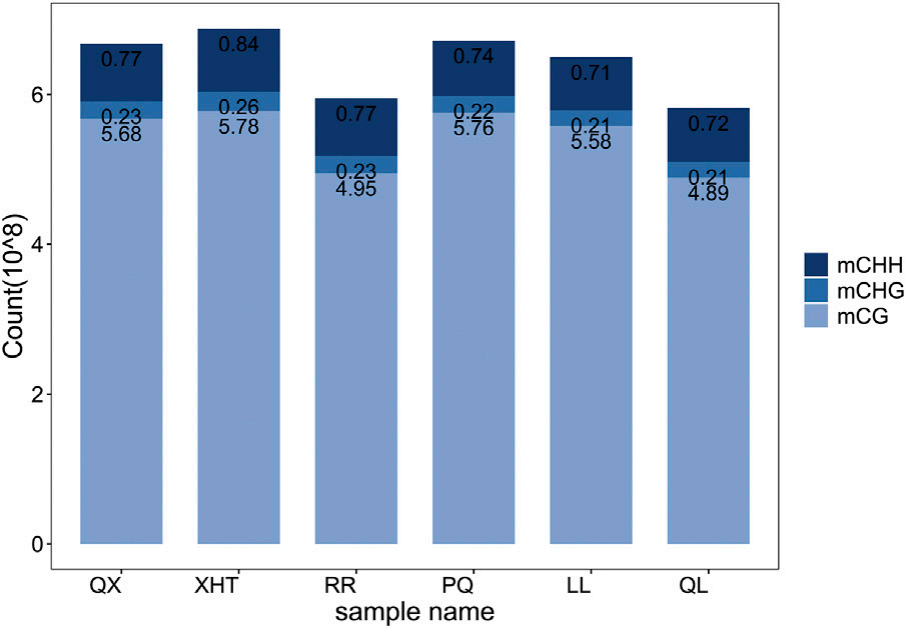
Frequency statistics of three sequence environments methylated cytosine. Different color of the bar represents the sequence environment of methylated cytosine.

By comparing the methylation data of six giant panda blood samples with the reference genome, about 60%–70% of clean reads were mapped to the giant panda reference genome (Table 1), and the data matching rate is relatively high (Ren et al., 2019). After comparing the results and extracting the statistics of methylation site information, we found that the total cytosine methylation ratio of all samples was between 5.35% and 5.49%. Consistent with other organisms, DNA cytosine methylation was present in three sequence environments: CG, CHG and CHH. Among the three sequence environments, the methylation ratio of the CG sequence was the highest, and the methylation ratios of the CHG and CHH sequences were far less than the CG sequence (Table 2, Figure 1). This suggests that the DNA methylation of giant panda occurs mainly at CpG dinucleotides, consistent with the methylation pattern of the mammalian C site (Head, 2014). Therefore, the subsequent analysis was performed mainly for the methylation sites in the CG sequence environment.

**TABLE 2:** Ratio statistics of genome cytosine methylation.

Sample	Qx	Xht	RR	PQ	LL	QL
Methylated C sites	667687248	687588186	594658757	671660061	650231100	582135310
Un-methylated C sites	11700079887	11846212052	10516549807	11757370201	11423526334	10126339596
Total C sites	12367767135	12533800238	11111208564	12429030262	12073757434	10708474906
Methylation ratio of C site	5.40%	5.49%	5.35%	5.40%	5.39%	5.44%
Methylated C sites in CG sequence	567751324	578483523	495273204	575718815	557911216	489209942
Un-methylated C sites in CG sequence	134356783	142470661	129100238	141419196	128842436	115048656
Total C sites in CG sequence	702108107	720954184	624373442	717138011	686753652	604258598
Methylation ratio of C site in CG sequence	80.86%	80.24%	79.32%	80.28%	81.24%	80.96%
Methylated C sites in CHG sequence	23148873	25521700	22715735	22043857	21004923	21299853
Un-methylated C sites in CHG sequence	2590704419	2644643902	2332157801	2625110648	2539644080	2260233698
Total C sites in CHG sequence	2613853292	2670165602	2354873536	2647154505	2560649003	2281533551
Methylation ratio of C site in CHG sequence	0.89%	0.96%	0.96%	0.83%	0.82%	0.93%
Methylated C sites in CHH sequence	76787051	83582963	76669818	73897389	71314961	71625515
Un-methylated C sites in CHH sequence	8975018685	9059097489	8055291768	8990840357	8755039818	7751057242
Total C sites in CHH sequence	9051805736	9142680452	8131961586	9064737746	8826354779	7822682757
Methylation ratio of C site in CHH sequence	0.85%	0.91%	0.94%	0.82%	0.81%	0.92%

Methylation ratio (%) = the number of methylation C sites in specific sequence environment/the total number of C sites * 100% 2 H represents A base or T base or C base

### 3.3 Screening and identification of DMGs

**FIGURE 2:**
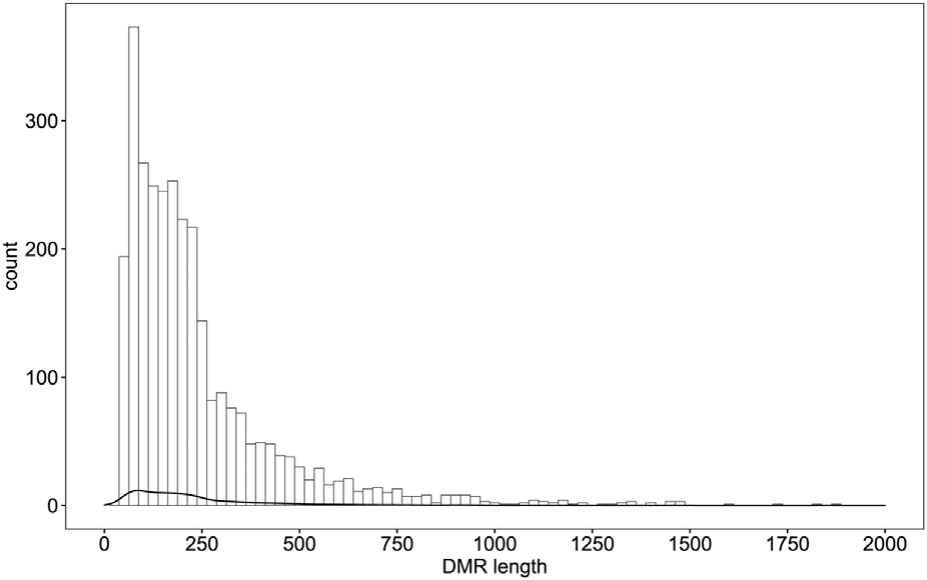
Distribution of DMRs in wildness training giant pandas compared with captive giant pandas. X axis indicates the counts of DMRs.

**FIGURE 3:**
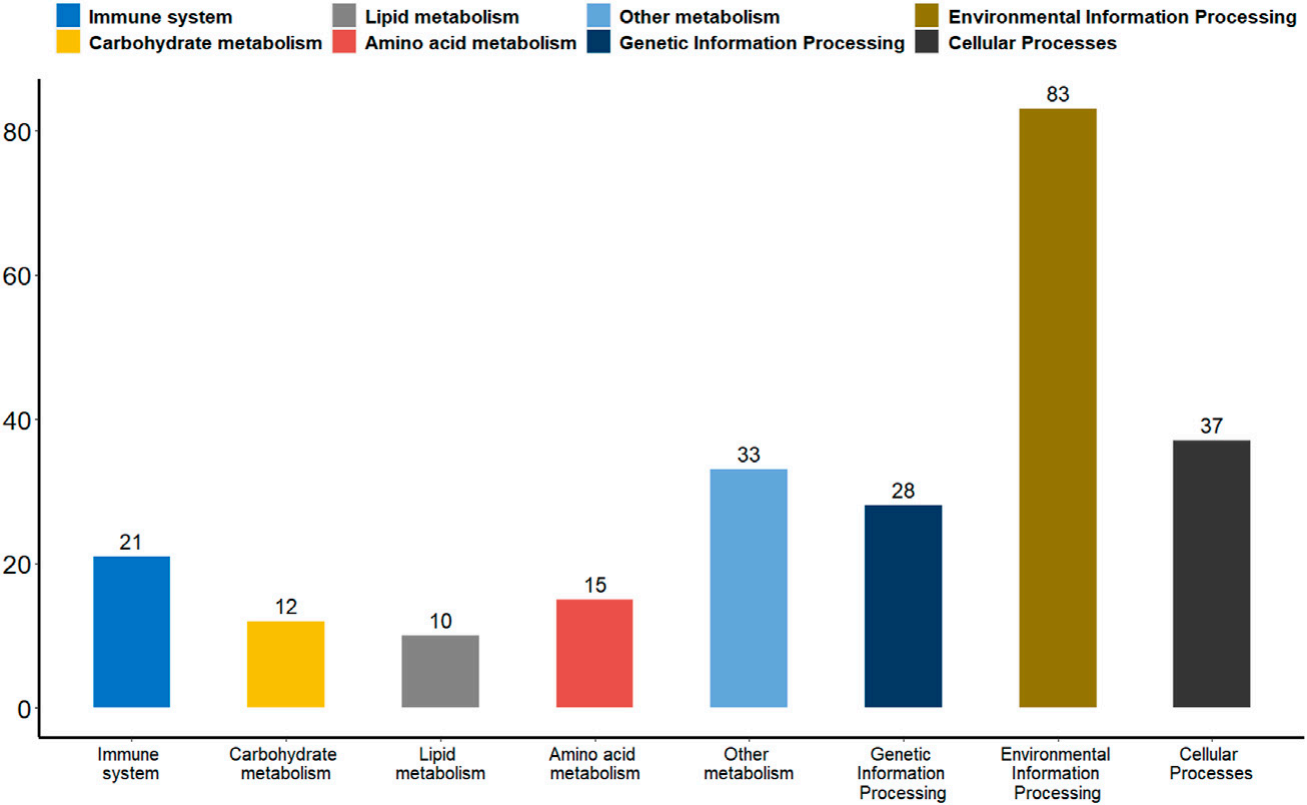
A bar plot of the functional annotation results of DMGs. The X-axis represents the biological process on the annotation. The *Y*-axis represents the number of DMGs for a biological process on the annotation. We extracted only the immune system, carbohydrate metabolism, lipid metabolism, amino acid metabolism, metabolism of other substances, genetic information processes, environmental information processes and cellular processes for our presentations.

Differential methylation analysis was performed using the DSS software package between the wild training group and the captive group, followed by extraction of differentially methylated regions. A total of 3,004 DMRs in the CG sequence environment were identified (Supplementsary Table S2). The length distribution ranged from 51bp to 2,433bp, with most being between 51 and 500bp, and the number of DMRs above 1,000bp was relatively small (Figure 2). Then, 3,004 DMRs were annotated to the giant panda reference genome using GenomicRanges software package. It was found that there were 34 promoters, 50 exons and 550 introns overlapping with DMRs (Supplementary Table S3). During the annotation process, we obtained 544 DMRs-related DMGs which included 21 genes related to the immune system, 12 genes related to carbohydrate metabolism, 10 genes related to lipid metabolism, 15 genes related to amino acid metabolism, 33 genes related to the metabolism of other substances, 28 genes involved in genetic information processes such as transcription, translation, replication and repair, 83 genes involved in environmental information processes such as membrane transport, signal transduction, signaling molecules and interaction, and 37 genes involved in cellular processes (Figure 3).

### 3.4 GO functional enrichment analysis of DMGs

**FIGURE 4:**
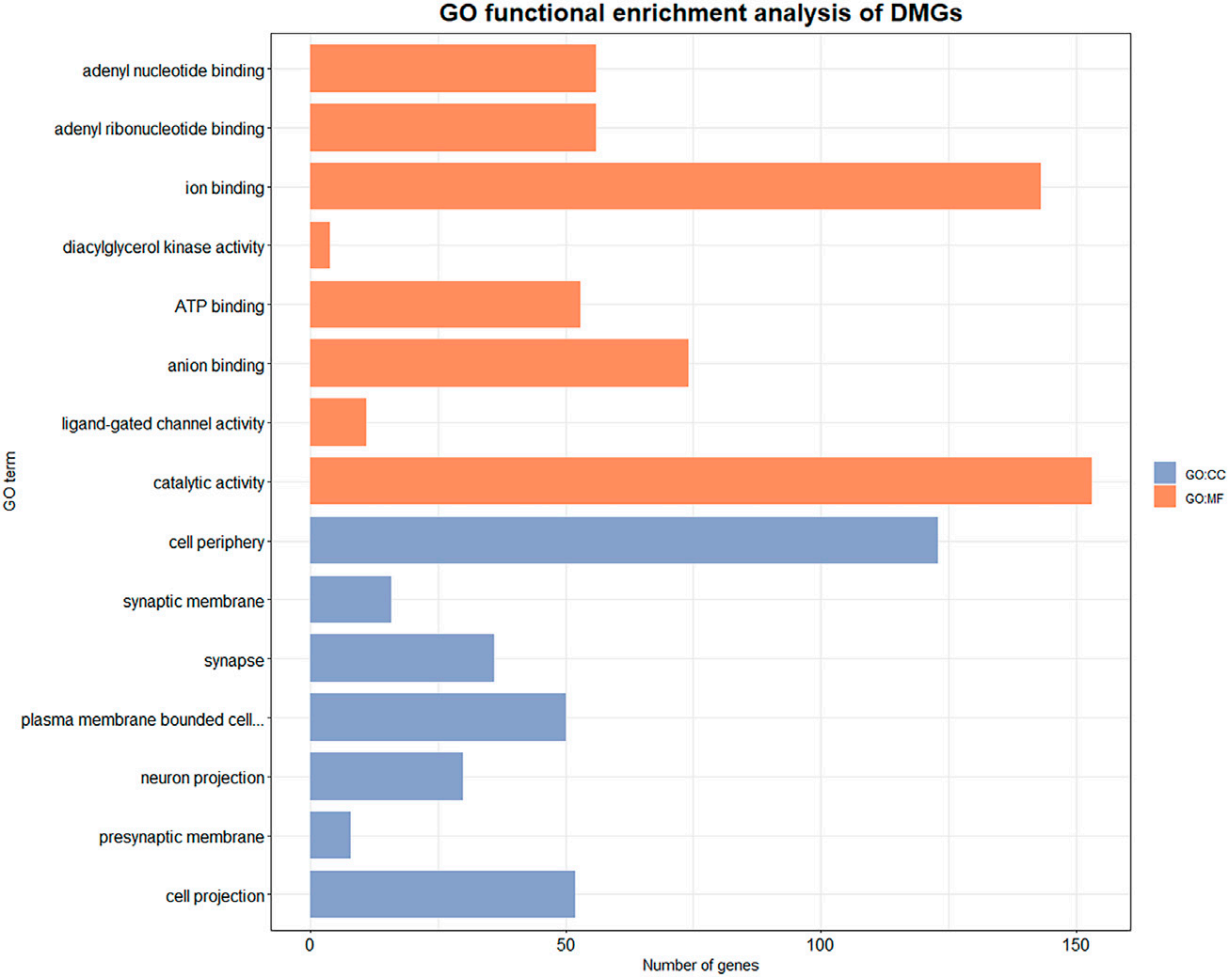
GO enrichment results of DMGs. Orange entries belong to molecular function, blue entries belong to cellular component. X axis indicates the number of genes involved in the GO enriched items, *Y* axis indicates the name of the item.

We performed GO function enrichment analysis on DMGs with a significance threshold set at 0.05. DMGs were significantly enriched in 75 GO entries, including 31 cell component (CC) entries and 44 molecular function (MF) entries (Supplementary Table S4 and Figure 4). The entries that are enriched in the cell component mainly included presynaptic membrane (GO:0042734), postsynaptic membrane (GO:0045211), postsynaptic density membrane (GO:0098839), synapse components such as postsynaptic specialization membrane (GO:0099634), and plasma membrane (GO:0005886), exocyst (GO:0000145), ion channel complex (GO: 0034702) and other components. The entries that are significantly enriched in molecular function mainly include ATP binding (GO:0005524), NAD + kinase activity (GO:0003951) and other energy utilization related entries, as well as diacylglycerol kinase activity (GO:0004143), phosphate hydrolase activity (GO:0042578), carbohydrate derivative binding (GO: 0097367) and other metabolism-related entries.

### 3.5 KEGG pathway enrichment analysis of DMGs

**FIGURE 5:**
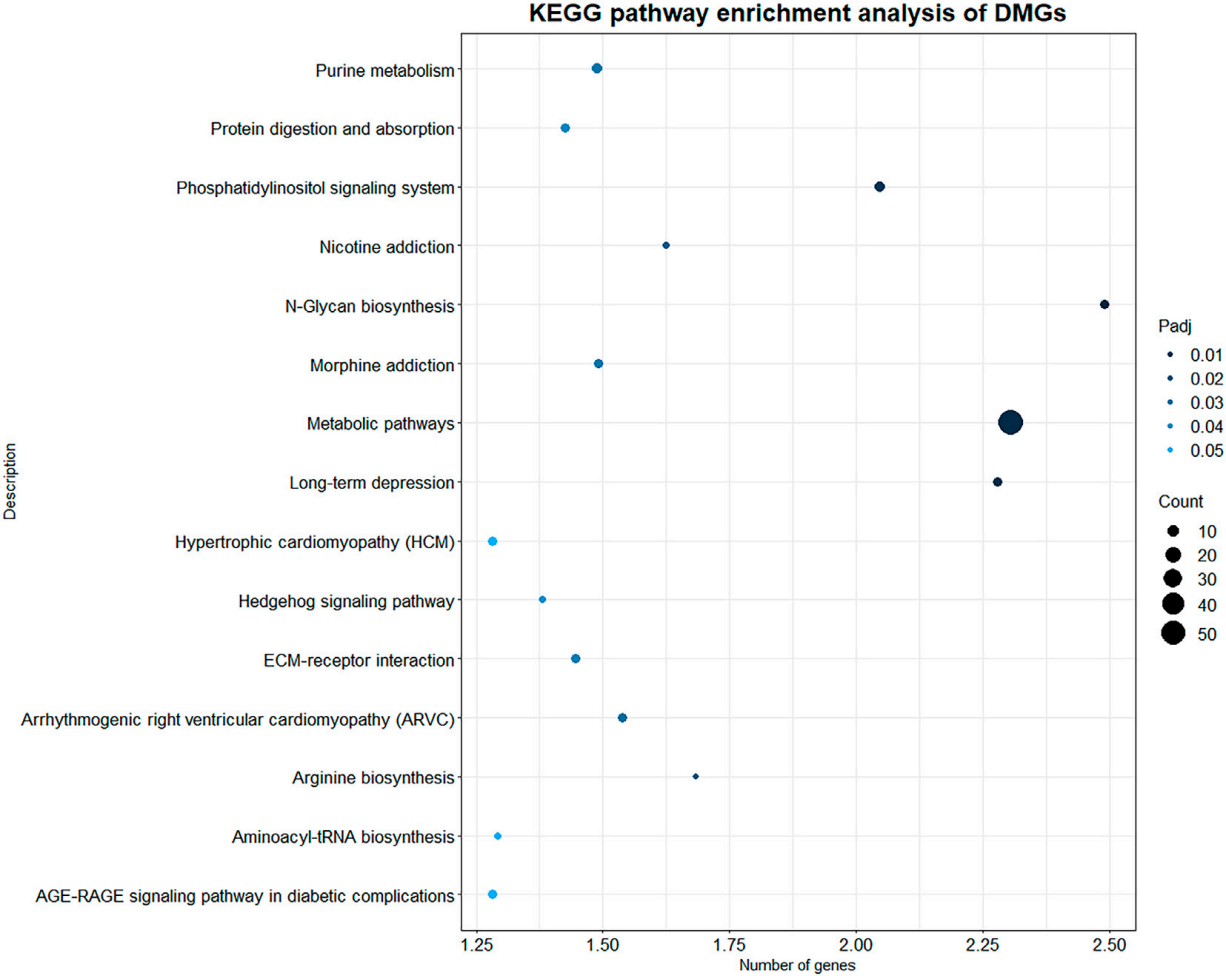
KEGG enrichment results of DMGs. X axis indicates the -log10(Padj) of KEGG enriched pathways, *Y* axis indicates the name of the pathway. The size of the circle represents the number of genes, and the color of the circle represents the *p*-value significance.

KEGG pathway enrichment analysis was performed with *p* value less than 0.05 as the statistical threshold, DMGs were enriched in a total of 243 pathways (Supplementary Table S5). Twelve of these pathways were significantly enriched, mainly including signaling pathways such as Phosphatidylinositol signaling system (aml04070), Hedgehog signaling pathway (aml04340); and metabolic pathways such as N-Glycan biosynthesis (aml00510), Purine metabolism (aml00230) (Figure 5). In the KEGG enrichment results, we also found some immune-related pathways, such as FcγR-mediated phagocytosis (aml04666), C-type lectin receptor signaling (aml04625), natural killer Mediated cytotoxicity (aml04650), chemokine signaling pathway (aml04062), Th17 cell differentiation (aml04659), Th1 and Th2 cell differentiation (aml04658) and so on. Immune-related DMGs VAV3, PLCG2, TEC, and PTPRC participated in multiple immune pathways. The DMGs involved in immune pathways and are shown in Table 3.

**TABLE 3:** Immune pathways and the enriched DMGs in KEGG pathway enrichment results.

Pathway	Gene id	Gene
Fc gamma R-mediated phagocytosis	ENSAMEG00000014051	*PLCG2*
	ENSAMEG00000001012	*VAV3*
	ENSAMEG00000012707	*PTPRC*
C-type lectin receptor signaling pathway	ENSAMEG00000011767	*ITPR2*
	ENSAMEG00000014051	*PLCG2*
	ENSAMEG00000003514	*MAPK10*
Leukocyte transendothelial migration	ENSAMEG00000000703	*CLDN1*
	ENSAMEG00000001012	*VAV3*
	ENSAMEG00000014051	*PLCG2*
B cell receptor signaling pathway	ENSAMEG00000001012	*VAV3*
	ENSAMEG00000014051	*PLCG2*
Natural killer cell mediated cytotoxicity	ENSAMEG00000001012	*VAV3*
	ENSAMEG00000014051	*PLCG2*
T cell receptor signaling pathway	ENSAMEG00000001012	*VAV3*
	ENSAMEG00000003081	*TEC*
	ENSAMEG00000012707	*PTPRC*
	ENSAMEG00000003514	*MAPK10*
NOD-like receptor signaling pathway	ENSAMEG00000011767	*ITPR2*
	ENSAMEG00000012726	*LOC100465205*
	ENSAMEG00000003514	*MAPK10*
Chemokine signaling pathway	ENSAMEG00000001012	*VAV3*
	ENSAMEG00000017944	*GRK3*
Th17 cell differentiation	ENSAMEG00000002899	*SMAD4*
	ENSAMEG00000003514	*MAPK10*
Cytosolic DNA-sensing pathway	ENSAMEG00000016022	*POLR3B*
RIG-I-like receptor signaling pathway	ENSAMEG00000003514	*MAPK10*
Th1 and Th2 cell differentiation	ENSAMEG00000003514	*MAPK10*
Toll-like receptor signaling pathway	ENSAMEG00000003514	*MAPK10*
IL-17 signaling pathway	ENSAMEG00000003514	*MAPK10*

The italic values in Table refer to the gene symbols corresponding to the gene ID.

### 3.6 Differentially methylated promoter analysis and the correlation analysis of differentially methylated promoter and gene expression

**FIGURE 6:**
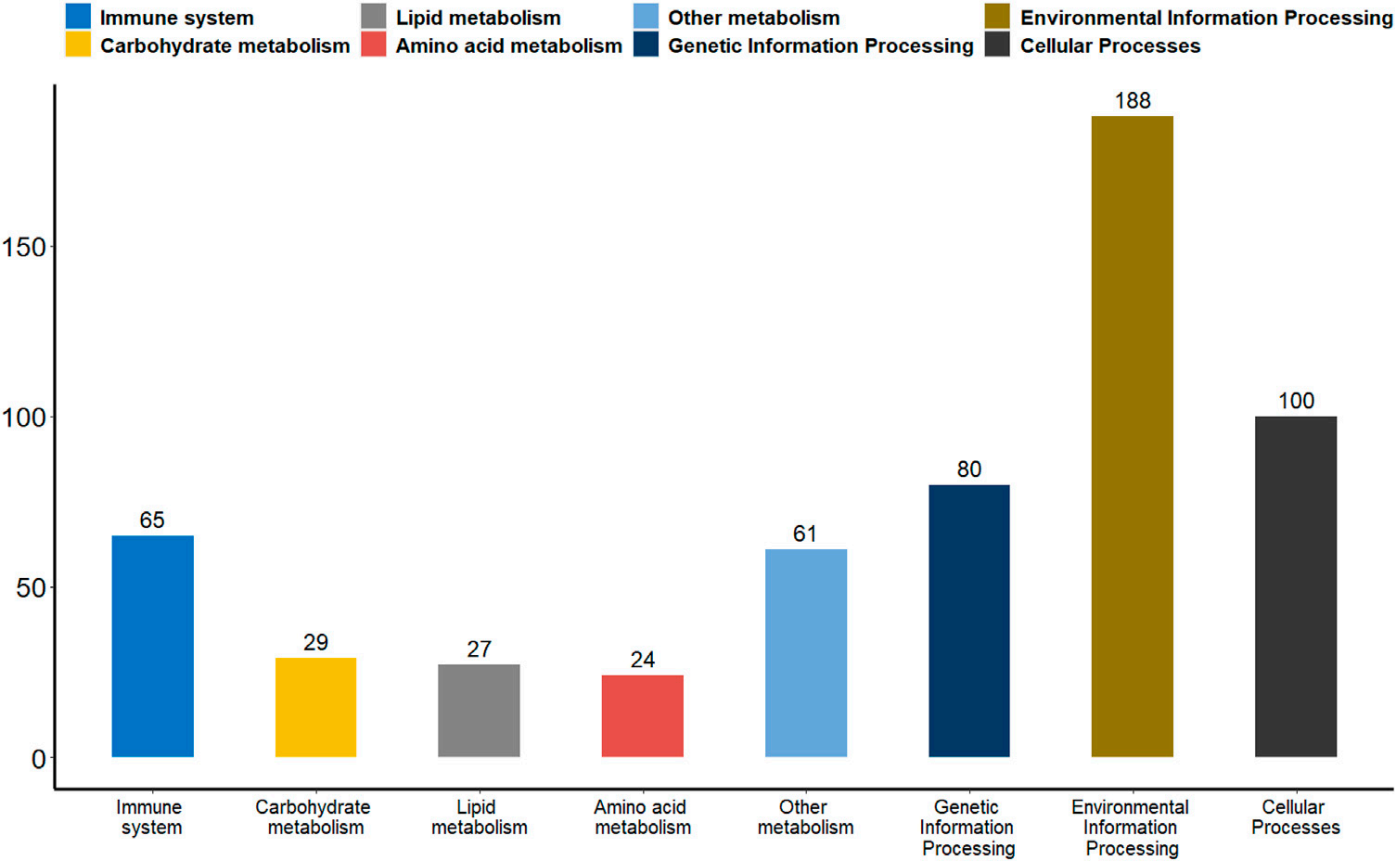
A bar plot of the functional annotation results of differentially methylated promoters. The X-axis represents the biological process on the annotation. The *Y*-axis represents the number of DMGs for a biological process on the annotation. We extracted only the immune system, carbohydrate metabolism, lipid metabolism, amino acid metabolism, metabolism of other substances, genetic information processes, environmental information processes and cellular processes for our presentations.

Promoter plays a key role in gene expression regulation (Deaton and Bird, 2011; Moore et al., 2013). We analyzed the promoter methylation levels of all genes between the two groups of giant pandas to understand whether there were differences in promoter methylation levels between wildness training giant pandas and captive giant pandas. With a *p*-value of 0.05 as the statistical threshold, a total of 1,199 differentially methylated promoters were identified. There were 325 hyper-methylated promoter genes and 874 hypo-methylated promoter genes in wildness training giant pandas compared to captive giant pandas. Among the differentially methylated promoters mentioned above, 65 genes were associated with the immune system, 29 genes with carbohydrate metabolism, 27 genes with lipid metabolism, 24 genes with amino acid metabolism, 61 genes with the metabolism of other substances, 80 genes involved in transcription, translation, replication and repair and other genetic information processes, 188 genes are involved in environmental information processes such as membrane transport, signal transduction, signaling molecules and interaction, and 100 genes are involved in cellular processes (Figure 6).

**FIGURE 7:**
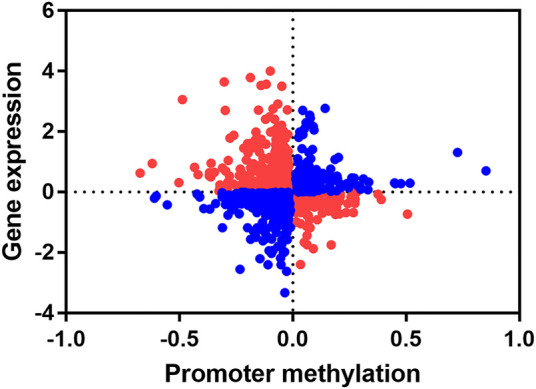
Conjoint analysis of promoter methylation level and gene expression level. Each dot represents a gene, red indicates that the promoter methylation level is negatively correlated with gene expression level, and blue indicates that the promoter methylation is positively correlated with gene expression level.

Since DNA methylation modifications in the promoter regions usually negatively regulate gene expression, we concentrate on genes with negative correlations between changes in methylation levels of promoter region and gene expression levels. We combined the promoter differential methylation data of 1,199 differentially methylated promoters with their transcriptome data. By calculating the Pearson correlation coefficient, we found a significant negative correlation between DNA methylation levels in promoters and gene expression levels (*r* = -0.05001, *p* < 0.05) (Figure 7). We found that there was an overlap between differentially methylated promoters and transcriptome differentially expressed genes (DEGs), and we extracted these genes. The hypo promoter methylation with up-regulated gene: *CCL5*, *ANP32A*, *MYOZ1*, *P2Y13*, *NME7*, *MRPS31*, *VWF*, *TPM1*, *PRKRIP1*, *GZMA*.

## 4 Discussion

Although epigenetic modification does not affect the change of a genome sequence, it can cause differences in phenotypes of organisms (D’Urso and Brickner, 2014). Increasing evidences suggest that DNA methylation is stable and heritable for the regulation of many life activities, such as the adaptation of organisms to their environment and the immune response of organisms (Huang et al., 2017; Rodriguez et al., 2017). Therefore, the construction of a genome-wide methylation map of giant panda can help to elucidate the methylation regulatory information of some important biological processes.

For the first time, we studied the differences in methylation of captive giant pandas with wildness training and without. The promoter is the core regulatory region for gene expression, and the methylation modification of the promoter region usually inhibits gene expression (Moore et al., 2013). We identified 1,199 differentially methylated promoters in differentially methylated promoter analysis. We found that *CCL5*, *P2Y13*, *GZMA*, *ANP32A*, *VWF*, *MYOZ1*, *NME7, MRPS31, TPM1* and *PRKRIP1* were up-regulated by hypomethylation of the promoter and these may play an important role in the environmental adaptation of giant pandas during wildness training. *CCL5* is an important immune-related gene and was one of the potential immunoassay markers in transcriptome analysis. It was also found in the DNA methylation expression patterns of peripheral monocytes of eight obese children with asthma and that the decrease in promoter methylation of *CCL5* was related to the occurrence of non-specific inflammation (Rastogi et al., 2013). *CCL5* plays an important role in innate immunity. Up-regulation of *CCL5* may help to enhance the innate immunity of giant pandas, which is important in the immune adaptation of pandas when undergoing wildness training. *P2Y* receptors are G protein-coupled receptors that act on purine and pyrimidine nucleotides. *P2Y13*’s endogenous ligand is ADP, and can be upregulated by type I interferon. Studies have shown that the ADP-mediated *P2Y12/P2Y13* signaling pathway protects the host against bacterial infections through ERK signaling (Zhang et al., 2018), and *P2Y13* and its ligand ADP can be released from infected cells as a signal during viral infection, limiting replication of DNA and RNA viruses (Zhang et al., 2019), having the potential as antiviral targets. *GZMA* encodes granzyme A. Granzyme and perforin can be released into the infected cells by natural killer cells and cytotoxic T lymphocytes to make them lyse, thereby eliminating pathogens when the body is infected (Dotiwala et al., 2016). *ANP32A*, also known as *pp32*, participates in the transcription of interferon-stimulated genes (*ISG*) induced by type I interferon (IFN) and contributes to the antiviral activity of cells (Kadota and Nagata, 2011). The up-regulation of these genes will help wildness training giant pandas to resist pathogen invasion, eliminate pathogen infection and enhance innate immune function. *VWF* is mainly derived from endothelial cells and is essential for the coagulation process (Kanaji et al., 2012). The coagulation of blood contributes to the healing of damaged wounds and is also an important immune defense barrier for the body to resist pathogen invasion in innate immunity (Esmon, 2004). The specific expression pattern of *VWF* in the wildness training giant panda may be caused by the increase in the probability of body damage when the body was exposed to branches, rocks, or even fighting with wild giant pandas or other animals in the wild environment. It may be the adaptation change to protect their health and stability. Myozenin (*MYOZ1* and *MYOZ3*) plays an important role in muscle fiber maturation. With the progress of muscle regeneration, the expression of *MYOZ1* gradually increases in mice (Yoshimoto et al., 2020). There were no artificial feeding conditions during the wildness training period, thus the training pandas needed to forage and find suitable food on a larger scale. The scope of their activities was significantly larger than captive giant pandas. Therefore, the high expression of *MYOZ1* assisted with increased exercise and ability to move through the wild habitat of giant pandas during wildness training. The expression trends of these genes indicated that giant pandas had strong immunity, exercise and blood coagulation abilities during wildness training. This may regulate the expression of corresponding genes through their negatively related promoter methylation to adapt to the wildness training process. *NME7* gene knockdown causes primary ciliary dyskinesia (Šedová et al., 2021), *MRPS31* gene deletion causes mitochondrial deregulation and the aggression of hepatocellular carcinoma (Min et al., 2021), and *TPM1* gene knockdown causes early embryonic death (Ma et al., 2021). The deletion or low expression of these genes above can cause disease development. All of these genes show a trend of low methylation and high expression during the wild training of giant pandas, which may be an adaptive strategy taken by giant pandas in the wild environment to counteract related diseases.

In addition to genes with differential methylation at promoter regions, we identified 544 DMGs from 1 kb upstream of the TSS to 1 kb downstream of the TTS. We conducted enrichment analysis of DMGs to better understand the physiological functions of differentially methylated modification. After GO functional enrichment, we found several items related to metabolism and synthesis processes such as ion channel activity and calcium ion binding. When environmental conditions change dramatically, organisms will develop a mechanism for selective accumulation and utilization of metal ions by changing the content of metal ions inside and outside the cell to adapt to the environment (Zambelli et al., 2012). Intracellular calcium is an important factor related to immune response and gene transcription (Li et al., 2013). For example, in immune cells, divalent cations such as calcium ions act as second messengers to regulate intracellular signaling pathways and immune responses (Feske et al., 2015). Additionally, calcium ions activate TCR-induced cell activation is also one of the keys to immune regulation. Stronger conditions are conducive to Th1 differentiation, while weaker Ca^2+^ signals are more biased towards Th2-type immunity (Hasso-Agopsowicz et al., 2018). At the same time, the calcium signal in lymphocytes regulate the activation and inhibition of T cells and B cells, and cellular immunity is mediated by cytotoxic T lymphocytes (Feske, 2007). Studies have shown that eukaryotic cells can sense metal ions at the pre-transcriptional level (Zambelli et al., 2012). Enrichment of a large number of DMGs into items such as ion channel activity and calcium ion binding may be related to the regulation of cellular immune activation during the wildness training of giant pandas. In the DNA methylation analysis of peripheral blood mononuclear cells of human infants with BCG vaccination, it was found that different DNA methylation patterns were also enriched in several immune pathways that directly or indirectly affect the immune response (Hasso-Agopsowicz et al., 2018). Similar to our conclusions and in addition to immune pathways, intracellular metabolism and synthesis process pathways such as potassium and calcium channels, and G protein-coupled receptors can drive vaccine-induced immune responses (Hasso-Agopsowicz et al., 2018). The results of KEGG pathway enrichment of DMGs demonstrated that the significantly enriched pathways of DMGs were related to synthesis and metabolism, such as glycan biosynthesis: N-glycan biosynthesis. Glycans, glycan-binding proteins and glycosylation play an important role in the body’s recognition of pathogens and resistance to pathogenic microorganisms from entering cells (Baum and Cobb, 2017). Wildness training giant pandas were exposed to more inflammatory factors and pathogenic microorganisms during the training process, and need activate stronger innate immunity to resist the invasion of pathogens. The enrichment of DMGs into the glycan synthesis pathway may be related to the regulation of pathogen recognition. At the same time, immune-related DMGs, such as *VAV3*, *PLCG2*, *TEC* and *PTPRC* participated in multiple immune pathways (Miller and Berg, 2002; Tybulewicz, 2005; Saunders et al., 2014; Magno et al., 2019). Most of these genes act on adaptive immunity and are involved in the development and activation of T cells, which may be related to the stronger cellular immunity of the wildness training pandas mentioned in transcriptome analysis.

In summary, we found that a large number of key genes involved in adaptive immunity and immune activation were significantly differentially methylated after wild training in giant pandas, which may play an important role in the wild adaptation of giant pandas. Moreover, the expression of *CCL5*, *P2Y13*, *GZMA*, *ANP32A*, *VWF*, *MYOZ1*, *NME7*, *MRPS31* and *TPM1* genes could be promoted by hypomethylation of their promoter regions during wild training, which in turn could enhance the immunity, hemagglutination, motility and disease resistance of wild training giant pandas. We are the first to describe the DNA methylation profile of giant panda blood tissue and our results indicated methylation modification is involved in the adaptation of captive giant pandas when undergoing wildness training. Our study also provided potential monitoring indicators for the successful reintroduction of valuable and threatened animals to the wild.

## Data Availability

The datasets presented in this study can be found in online repositories. The names of the repository/repositories and accession number(s) can be found below: https://www.ncbi.nlm.nih.gov/, PRJNA857106; https://www.ncbi.nlm.nih.gov/, PRJNA878951.

## References

[B1] Allard S. Fuller G. Torgerson-White L. Starking M. D. Yoder-Nowak T. (2019). Personality in zoo-hatched Blanding’s turtles affects behavior and survival after reintroduction into the wild. Front. Psychol. 10, 2324. 10.3389/fpsyg.2019.02324 31681114 PMC6813202

[B2] Anastasiadi D. Díaz N. Piferrer F. (2017). Small ocean temperature increases elicit stage-dependent changes in DNA methylation and gene expression in a fish, the European sea bass. Sci. Rep. 7 (1), 12401–12412. 10.1038/s41598-017-10861-6 28963513 PMC5622125

[B3] Anderson O. S. Sant K. E. Dolinoy D. C. (2012). Nutrition and epigenetics: An interplay of dietary methyl donors, one-carbon metabolism and DNA methylation. J. Nutr. Biochem. 23 (8), 853–859. 10.1016/j.jnutbio.2012.03.003 22749138 PMC3405985

[B4] Baum L. G. Cobb B. A. (2017). The direct and indirect effects of glycans on immune function. Glycobiology 27 (7), 619–624. 10.1093/glycob/cwx036 28460052

[B5] Deaton A. M. Bird A. (2011). CpG islands and the regulation of transcription. Genes. Dev. 25 (10), 1010–1022. 10.1101/gad.2037511 21576262 PMC3093116

[B6] Dotiwala F. Mulik S. Polidoro R. B. Ansara J. A. Burleigh B. A. Walch M. (2016). Killer lymphocytes use granulysin, perforin and granzymes to kill intracellular parasites. Nat. Med. 22 (2), 210–216. 10.1038/nm.4023 26752517 PMC7325279

[B7] D’Urso A. Brickner J. H. (2014). Mechanisms of epigenetic memory. Trends Genet. 30 (6), 230–236. 10.1016/j.tig.2014.04.004 24780085 PMC4072033

[B8] Esmon C. T. (2004). Interactions between the innate immune and blood coagulation systems. Trends Immunol. 25 (10), 536–542. 10.1016/j.it.2004.08.003 15364056 PMC7185622

[B9] Feng H. Conneely K. N. Wu H. (2014). A Bayesian hierarchical model to detect differentially methylated loci from single nucleotide resolution sequencing data. Nucleic Acids Res. 42 (8), e69. 10.1093/nar/gku154 24561809 PMC4005660

[B10] Feske S. (2007). Calcium signalling in lymphocyte activation and disease. Nat. Rev. Immunol. 7 (9), 690–702. 10.1038/nri2152 17703229

[B11] Feske S. Wulff H. Skolnik E. Y. (2015). Ion channels in innate and adaptive immunity. Annu. Rev. Immunol. 33, 291–353. 10.1146/annurev-immunol-032414-112212 25861976 PMC4822408

[B12] Goldberg A. D. Allis C. D. Bernstein E. (2007). Epigenetics: A landscape takes shape. Cell. 128 (4), 635–638. 10.1016/j.cell.2007.02.006 17320500

[B13] Han B. Li W. Chen Z. Xu Q. Luo J. Shi Y. (2016). Variation of DNA methylome of zebrafish cells under cold pressure. PloS one 11 (8), e0160358. 10.1371/journal.pone.0160358 27494266 PMC4975392

[B14] Hasso-Agopsowicz M. Scriba T. J. Hanekom W. A. Dockrell H. M. Smith S. G. (2018). Differential DNA methylation of potassium channel KCa3. 1 and immune signalling pathways is associated with infant immune responses following BCG vaccination. Sci. Rep. 8 (1), 13086–13111. 10.1038/s41598-018-31537-9 30166570 PMC6117309

[B15] Head J. A. (2014). Patterns of DNA methylation in animals: An ecotoxicological perspective. Integr. Comp. Biol. 54 (1), 77–86. 10.1093/icb/icu025 24785828

[B16] Hu J. Askary A. M. Thurman T. J. Spiller D. A. Palmer T. M. Pringle R. M. (2019). The epigenetic signature of colonizing new environments in Anolis Lizards. Mol. Biol. Evol. 36 (10), 2165–2170. 10.1093/molbev/msz133 31147693

[B17] Huang X. Li S. Ni P. Gao Y. Jiang B. Zhou Z. (2017). Rapid response to changing environments during biological invasions: DNA methylation perspectives. Mol. Ecol. 26 (23), 6621–6633. 10.1111/mec.14382 29057612

[B18] Kadota S. Nagata K. (2011). pp32, an INHAT component, is a transcription machinery recruiter for maximal induction of IFN-stimulated genes. J. Cell. Sci. 124 (6), 892–899. 10.1242/jcs.078253 21325029

[B19] Kanaji S. Fahs S. A. Shi Q. Haberichter S. L. Montgomery R. (2012). Contribution of platelet vs. endothelial VWF to platelet adhesion and hemostasis. J. Thromb. Haemost. 10 (8), 1646–1652. 10.1111/j.1538-7836.2012.04797.x 22642380 PMC3419786

[B20] Krueger F. Andrews S. R. (2011). Bismark: A flexible aligner and methylation caller for bisulfite-seq applications. bioinformatics 27 (11), 1571–1572. 10.1093/bioinformatics/btr167 21493656 PMC3102221

[B21] Lawrence M. Huber W. Pages H. Aboyoun P. Carlson M. Gentleman R. (2013). Software for computing and annotating genomic ranges. PLoS Comput. Biol. 9 (8), e1003118. 10.1371/journal.pcbi.1003118 23950696 PMC3738458

[B22] Li S. Y. Du M. J. Wan Y. J. Lan B. Liu Y. H. Yang Y. (2013). The N-terminal 20-amino acid region of guanine nucleotide exchange factor Vav1 plays a distinguished role in T cell receptor-mediated calcium signaling. J. Biol. Chem. 288 (6), 3777–3785. 10.1074/jbc.M112.426221 23271736 PMC3567632

[B23] Liu Q. A. (2013). The impact of climate change on plant epigenomes. Trends Genet. 29 (9), 503–505. 10.1016/j.tig.2013.06.004 23806639

[B24] Ma S. Xu Q. Bai R. Dong T. Peng Z. Liu X. (2021). Generation of a TPM1 homozygous knockout embryonic stem cell line by CRISPR/Cas9 editing. Stem Cell. Res. 55, 102470. 10.1016/j.scr.2021.102470 34352617

[B25] Maghbooli Z. Hossein-Nezhad A. Adabi E. Asadollah-Pour E. Sadeghi M. Mohammad-Nabi S. (2018). Air pollution during pregnancy and placental adaptation in the levels of global DNA methylation. PLoS One 13 (7), e0199772. 10.1371/journal.pone.0199772 29979694 PMC6034814

[B26] Magno L. Lessard C. B. Martins M. Lang V. Cruz P. Asi Y. (2019). Alzheimer’s disease phospholipase C-gamma-2 (PLCG2) protective variant is a functional hypermorph. Alzheimers Res. Ther. 11 (1), 16–11. 10.1186/s13195-019-0469-0 30711010 PMC6359863

[B27] Martin-Wintle M. S. Kersey D. C. Wintle N. J. Aitken-Palmer C. Owen M. A. Swaisgood R. R. (2019). Comprehensive breeding techniques for the giant panda. Adv. Exp. Med. Biol. 1200, 275–308. 10.1007/978-3-030-23633-5_10 31471801

[B28] Miller A. T. Berg L. J. (2002). New insights into the regulation and functions of Tec family tyrosine kinases in the immune system. Curr. Opin. Immunol. 14 (3), 331–340. 10.1016/s0952-7915(02)00345-x 11973131

[B29] Min S. Lee Y. K. Hong J. Park T. J. Woo H. G. Kwon S. M. (2021). MRPS31 loss is a key driver of mitochondrial deregulation and hepatocellular carcinoma aggressiveness. Cell. Death Dis. 12 (11), 1076–1112. 10.1038/s41419-021-04370-8 34772924 PMC8589861

[B30] Moore L. D. Le T. Fan G. (2013). DNA methylation and its basic function. Neuropsychopharmacology 38 (1), 23–38. 10.1038/npp.2012.112 22781841 PMC3521964

[B31] Rastogi D. Suzuki M. Greally J. M. (2013). Differential epigenome-wide DNA methylation patterns in childhood obesity-associated asthma. Sci. Rep. 3 (1), 2164–2211. 10.1038/srep02164 23857381 PMC3712321

[B32] Ren J. Shen F. Zhang L. Sun J. Yang M. Yang M. (2019). Single-base-resolution methylome of giant panda’s brain, liver and pancreatic tissue. PeerJ 7, e7847. 10.7717/peerj.7847 31637123 PMC6800980

[B33] Rodriguez R. M. Suarez-Alvarez B. Lavín J. L. Mosén-Ansorena D. Raneros A. B. Márquez-Kisinousky L. (2017). Epigenetic networks regulate the transcriptional program in memory and terminally differentiated CD8+ T cells. J. Immunol. 198 (2), 937–949. 10.4049/jimmunol.1601102 27974453

[B34] Sagonas K. Meyer B. S. Kaufmann J. Lenz T. L. Hasler R. Eizaguirre C. (2020). Experimental parasite infection causes genome-wide changes in DNA methylation. Mol. Biol. Evol. 37 (8), 2287–2299. 10.1093/molbev/msaa084 32227215 PMC7531312

[B35] Saunders A. E. Shim Y. A. Johnson P. (2014). Innate immune cell CD45 regulates lymphopenia-induced T cell proliferation. J. Immunol. 193 (6), 2831–2842. 10.4049/jimmunol.1302681 25114101

[B36] Šedová L. Buková I. Bažantová P. Petrezsélyová S. Prochazka J. Školníková E. (2021). Semi-lethal primary ciliary dyskinesia in rats lacking the nme7 gene. Int. J. Mol. Sci. 22 (8), 3810. 10.3390/ijms22083810 33916973 PMC8067621

[B37] Shi K. Ge M. N. Chen X. Q. (2020). Coordinated DNA methylation and gene expression data for identification of the critical genes associated with childhood atopic asthma. J. Comput. Biol. 27 (1), 109–120. 10.1089/cmb.2019.0194 31460781

[B38] Shi Y. Y. Huang Z. Y. Zeng Z. J. Wang Z. L. Wu X. B. Yan W. Y. (2011). Diet and cell size both affect queen-worker differentiation through DNA methylation in honey bees (*Apis mellifera*, Apidae). PloS one 6 (4), e18808. 10.1371/journal.pone.0018808 21541319 PMC3082534

[B39] Tammen S. A. Friso S. Choi S. W. (2013). Epigenetics: The link between nature and nurture. Mol. Asp. Med. 34 (4), 753–764. 10.1016/j.mam.2012.07.018 PMC3515707 22906839

[B40] Thiebaut F. Hemerly A. S. Ferreira P. C. G. (2019). A role for epigenetic regulation in the adaptation and stress responses of non-model plants. Front. Plant Sci. 10, 246. 10.3389/fpls.2019.00246 30881369 PMC6405435

[B41] Tönges S. Venkatesh G. Andriantsoa R. Hanna K. Gatzmann F. Raddatz G. (2021). Location-dependent DNA methylation signatures in a clonal invasive crayfish. Front. Cell. Dev. Biol. 9:794506. 10.3389/fcell.2021.794506 34957121 PMC8695926

[B42] Tybulewicz V. L. (2005). Vav-family proteins in T-cell signalling. Curr. Opin. Immunol. 17 (3), 267–274. 10.1016/j.coi.2005.04.003 15886116

[B43] Waterland R. A. (2006). Epigenetic mechanisms and gastrointestinal development. J. Pediatr. (N. Y., N. Y. U. S.) 149 (5), S137–S142. 10.1016/j.jpeds.2006.06.064 17212956

[B44] Wei F. Swaisgood R. Hu Y. Nie Y. Yan L. Zhang Z. (2015). Progress in the ecology and conservation of giant pandas. Conserv. Biol. 29 (6), 1497–1507. 10.1111/cobi.12582 26372302

[B45] Xiaoping T. A. N. G. Jiansheng J. I. A. Zhichen W. A. N. G. Dehui Z. H. A. N. G. Baocheng Y. U. Jianbing Y. U. (2015). Scheme design and main result analysis of the fouth national survey on giant pandas. For. Resour. WANAGEMENT 0 (1), 11. 10.13466/j.cnki.lyzygl.2015.01.002

[B46] Yang M. Huang Y. Wu H. Li C. Ling S. Sun J. (2022). Blood transcriptome analysis revealed the immune changes and immunological adaptation of wildness training giant pandas. Mol. Genet. Genomics. 297 (1), 227–239. 10.1007/s00438-021-01841-7 34985592

[B47] Yoshimoto Y. Ikemoto-Uezumi M. Hitachi K. Fukada S. I. Uezumi A. (2020). Methods for accurate assessment of myofiber maturity during skeletal muscle regeneration. Front. Cell. Dev. Biol. 8, 267. 10.3389/fcell.2020.00267 32391357 PMC7188918

[B48] Zambelli B. Musiani F. Ciurli S. (2012). Metal ion-mediated DNA-protein interactions. Mater. Ions Life Sci. 10, 135–170. 10.1007/978-94-007-2172-2_5 22210338

[B49] Zhang C. Yan Y. He H. Wang L. Zhang N. Zhang J. (2019). IFN-stimulated P2Y13 protects mice from viral infection by suppressing the cAMP/EPAC1 signaling pathway. J. Mol. Cell. Biol. 11 (5), 395–407. 10.1093/jmcb/mjy045 30137373 PMC7107496

[B50] Zhang X. Qin J. Zou J. Lv Z. Tan B. Shi J. (2018). Extracellular ADP facilitates monocyte recruitment in bacterial infection via ERK signaling. Cell. Mol. Immunol. 15 (1), 58–73. 10.1038/cmi.2016.56 27867196 PMC5827171

[B51] Zhang Z. Zhang S. Wei F. Wang H. Ming L. I. Jinchu H. U. (2006). Translocation and discussion on reintroduction of captive giant panda. Acta Theriol. Sin. 26, 292–299.

